# Spatial patterns of foreign agricultural land holdings near U.S. military interest points

**DOI:** 10.1371/journal.pone.0355827

**Published:** 2026-08-12

**Authors:** Valerie Kilders, Anam Ali, Austin Berenda, Michael Smith, Nicole Olynk Widmar, Ronald F. Turco

**Affiliations:** 1 Department of Agricultural Economics, Purdue University, West Lafayette, Indiana, United States of America; 2 Department of Agronomy, Purdue University, West Lafayette, Indiana, United States of America; UF: University of Florida, UNITED STATES OF AMERICA

## Abstract

Foreign holdings of U.S. farmland have received increased regulatory and public attention amid concerns about national security risks, particularly when adversary-linked acquisitions occur near military installations. Most existing research focuses on the volume rather than the spatial distribution of these holdings. Using social media listening, we document a sharp rise in public discussion of foreign agricultural land holdings since 2020, coinciding with the post-2022 wave of state-level legislation. We then assess the spatial relationship between foreign land transactions and U.S. Military Interest Points (MIPs) using transaction-level data from the Agricultural Foreign Investment Disclosure Act covering 1996–2024. People’s Republic of China (PRC)-linked transactions excluding the 2013 Smithfield acquisition occur in MIP counties at 43.7%, compared with approximately 11% for non-adversary foreign and unidentified-origin transactions. Linear probability and probit specifications with state and acquisition-year fixed effects estimate that MIP presence is associated with a 0.47 percentage-point to 0.75 percentage point higher probability that a transaction is PRC- linked, while non-adversary foreign transactions show no MIP association. Descriptive evidence suggests that PRC-linked transactions shifted from MIP counties toward MIP-adjacent counties after 2022, while the share of transactions reported under unidentified-origin codes rose in both MIP and MIP-adjacent counties, though small post-2022 sample sizes preclude statistical inference. Our findings are associational rather than causal and inform ongoing policy discussions about foreign agricultural land ownership.

## 1. Introduction

The United States of America (U.S.) has about 880 million acres of farmland [1]. A little more than 46 million (5.2%) of this total acreage is owned or leased by foreign countries including Canada, the Netherlands, Germany, and The People’s Republic of China (henceforth referred to as PRC) [2]. While often downplayed or referenced as a small share of the overall farmland [3] foreign-holdings have increased nearly 2.2 million acres (0.25%) annually since 2015 [4]. The strategic value of the location of land holdings relative to total area acquired is rarely considered.

The uniqueness of land as an asset has long positioned a debate about whether foreign land purchases are different (or should be treated differently) than other foreign investments [5]. At the same time, policymakers have voiced concerns about agricultural land being controlled by foreign entities and introduced or even passed various pieces of legislation aiming to “protect American Farmland” [6]. Indeed, starting in 2022 a wave of legislation took place with 22 States introducing and/or passing legislation restricting and limiting the acquisition of land by foreign entities [7]. A significant share of this legislation actively targets foreign adversaries such as the PRC. However, whether these foreign control efforts are a reflection of their voters’ concern, i.e., the public, has not yet been explored. Recent work by Lin and Ortega [8] finds that state-level legislative action is driven by national security concerns and political ideology, with the presence of military installations closely linked to legislative activity. However, whether the underlying spatial pattern of foreign acquisitions itself, particularly by adversarial nations, matches this legislative focus on military proximity has not yet been empirically documented.

Our study assesses whether policymakers’ increased attention to this matter is reflected in the public discourse. Following previous studies, e.g., [9–11] we use social media listening to investigate how public attention towards foreign land holdings has developed in recent years. To reflect the public’s volume of interest, we focus on the total number of posts (i.e., mentions) about this topic from Mid 2020-Mid 2024. Doing so allows us to evaluate whether policymakers’ increased focus on this topic matches voters’ growing interest.

Beyond public interest, a subset of studies has looked at land ownership or leasing by countries deemed adversarial by the U.S. government, such as the PRC [12,13]. Collectively, they find that adversarial countries only own or lease a small share of land in the U.S. For example, as of 2024, the PRC only held about 247,659 acres which equates to less than 0.6% of foreign-held acres [2]. These findings have led researchers to conclude that policymakers’ concerns are not proportionate to the extent of the perceived risk [3].

However, as acknowledged in the 2024 AFIDA report itself [2], not only should reported numbers be considered as a minimum: when adversarial investors operate through multi-country investment groups or complex organizational structures, current reporting cannot accurately capture these holdings [2]. Adversarial interest may thus be underrepresented. Further, small absolute values do not eliminate the possibility of concern or threat. Most assessments thus far have purely focused on the quantity of land owned or leased by foreign or even hostile nations. Relatively little attention has been paid to where exactly those relatively few landholdings are in relation to other assets or points of interest. One particular concern that has been raised is the purchase of agricultural land near military interest points (MIP) in the U.S. [14,15]. Concerns are that purchasing land within immediate proximity to MIPs could pose a threat to national security [16]. While anecdotal evidence of foreign land holdings near MIPs has been reported [17], systematic empirical analysis of the extent and pattern of such acquisitions by adversarial countries has not been conducted.

Our study addresses this gap between anecdotal evidence of foreign land holdings near MIPs and empirical analyses. We combine transaction-level data from the Agricultural Foreign Investment Disclosure Act (AFIDA) [18] with geographic data from the Committee on Foreign Investment in the United States (CFIUS) Part 802 [19] and the 2020 Census to identify counties containing or adjacent to points of U.S. military interest. We pair these with county-level agricultural land value data from Acres [20]. Because non-PRC adversary transactions are too few in the AFIDA data to support separate analysis (fewer than 15 transactions across Iran, North Korea, and Russia combined for the entire study period), our empirical focus is on PRC-linked transactions specifically, with non-adversary foreign transactions serving as a placebo comparison. We also examine transactions recorded under unidentified country codes (AFIDA codes 998 and 999), which capture opaque ownership structures that could mask adversarial involvement.

Our analysis documents the share of foreign holdings in MIP counties and in counties adjacent to MIPs, and tests whether PRC-linked transactions are systematically more concentrated near MIPs than other foreign transactions. We further examine whether this concentration pattern appears to have shifted after the 2022 legislative wave, though the small number of post-2022 adversary transactions limits this analysis to descriptive and directional evidence rather than formal statistical testing.

We provide the first systematic empirical evidence of the spatial relationship between PRC-linked land transactions and U.S. military infrastructure. Assisted through a placebo test, we establish that this concentration is specific to PRC acquisitions rather than a general feature of foreign land purchases. Further, we offer directional descriptive evidence on how spatial patterns evolved following the 2022 legislative wave. We then discuss the results relative to the quality and availability of current data.

Our results contribute to informing the public discourse on foreign land holdings and inform policy discussions by incorporating agricultural land values alongside national security considerations. In doing so, we offer a more complete look into the explanations for land purchases.

## 2. Methods

### 2.1. Collection of social media listening data

To better understand public attention surrounding foreign land ownership by malign foreign nations, we employ social media listening. This technique effectively harnesses online data, from news sources as well as a variety of blogs, commentaries, and social networking platforms, offering observations of public opinions in a way that avoids the potential biases of survey data [21]. Furthermore, online and social media data can be collected over broad ranges of time to construct a time series dataset. Many techniques exist for extracting online data; we employ the Quid platform (previously known as Netbase), which also provides access to natural language processing technology to analyze posts [22].

The Quid platform enables researcher-parameterized searches of online and social media through the construction of a series of inclusive and exclusive search keywords. Additional parameters for data collection are applied to limit the spatial and temporal range of data collected. Effectively, we collected conversations (or mentions of our inclusive search terms) that match our keywords, found in posts occurring in the United States and minor outlying islands between 06/01/2020 and 08/31/2024. In addition to the inclusive search terms, exclusionary terms can be used to eliminate or remove media from the search results through a tuning process. These removals are conducted to avoid cases where a post is a false match for a keyword, usually due to slang, misspellings, and homonyms. Primary inclusionary search keywords and excluded terms are displayed in Tables A1 and A2 in the S1 Appendix in S1 File. Search results were collected in the week of 09/09/2024 from a variety of platforms, primarily Twitter (now X) and news feeds with the main domains reported in Table A3 in the S1 Appendix in S1 File. The corresponding data are reported in the S2 Appendix. The search results do not include Facebook or Instagram (due to web scraping restrictions [23,24]), but the available sources allow us to capture the general public trend [25].

It should be noted that our analysis focuses on capturing general discourse created either organically or through active promotion of the issues. As such posts are analyzed by examining counts on a weekly basis, independent of the motivation behind the posts.

### 2.2. Collection of public land holding and MIP data

To determine whether land owned or leased by investors from adversarial countries is located in specific areas for statistically identifiable reasons, such as favorable land values or proximity to U.S. MIPs, we combine several publicly available datasets into a transaction-level cross-sectional dataset. Each observation represents a single foreign agricultural land transaction and includes the acquiring entity's country of origin, the parcel's location and acreage, the purchase price, the closest-available land value, and the transaction year. The dataset is restricted to counties that experienced at least one foreign transaction during 1996–2024. To contextualize the observed spatial distribution of foreign transactions against the geographic footprint of military infrastructure, we supplement our analysis with a county-year panel covering all U.S. counties from 1996 through 2024. This panel is used only to compute the share of all U.S. counties that contain or are adjacent to MIPs and is not used for regression analysis. Given the rarity of PRC and other adversary transactions, combined with the near-time-invariance of MIP status within the sample window, the dataset leaves too few county-year observations with positive outcomes to support panel analyses.

Serving as the foundation for both data sets, we employed the 2020 U.S. census data [26] to create a list of all counties in the U.S. We then paired this information with county-level landholding data from AFIDA. The AFIDA data reports detailed information about foreign land ownership or leasing in each county, the year of investment and the country of origin (citizenship and/or permanent residency when it is an individual and principal place of business when it is an entity) of the investor (when available). We restrict the analysis to 1996–2024. The lower bound is determined by land value data availability, as our primary control variable is not available prior to 1996 [27,28]. This window also falls within a reasonably stable modern institutional environment for foreign agricultural investment and provides roughly 25 years of pre-period observations before the 2022 legislative wave.

We then created several dummy variables specific to the country of origin of the investor or investor group. Specifically, we constructed an indicator for PRC-linked acquisitions (*PRC = 1, 0 otherwise*) and a parallel indicator which excludes transactions associated with the 2013 Smithfield acquisition (*PRC excl. Smithfield* = *1, 0 otherwise*) [29]. The acquisition and its subsequent associated expansions account for 60 individual transactions, which is a substantial share of PRC-linked activity. As such, we treat these separately given that this single corporate acquisition drives a disproportionate share of PRC-coded transactions, which could obscure the broader pattern of PRC-linked land activity that is the focus of our analysis. An acquisition was linked to the PRC if the recorded interest was of a primary or secondary nature. Following the 2024 GAO report [30], USDA began capturing secondary and higher-order interests (beyond the primary investor) associated with China, Iran, North Korea, and Russia [2].

We also generated indicators for other adversarial countries as identified in Executive Order 13981 [31] and 15 CFR 791.4 [32] as of the time of data collection in the Fall of 2024; the list includes China (People’s Republic of), Hong Kong, Macau, Iran (the Islamic Republic of), Korea (Democratic People’s Republic of) and the Russian Federation (Venezuela was not included, as only politician Nicolás Maduro and internal regime allies were specified as adversaries). However, as indicated in the AFIDA dataset as well as the corresponding report [2], the attributable acquisitions are negligible (<15 transactions between 1996 and 2024). We thus focus our subsequent analysis solely on the PRC as an adversary of the U.S. Next, we generated an indicator for transactions in which no predominant country could be identified (code 999 in the AFIDA dataset) or in which no foreign investor was listed despite a reported foreign acquisition (code 998 in the AFIDA dataset) (*Unidentified = 1, 0 otherwise*).

We then extended the datasets further by adding county level data indicating the presence of MIPs throughout the U.S. To identify the location of MIPs, we used the interactive map of military installation provided by CFIUS Part 802 [33] to mark counties as either containing a MIP (*MIP = 1, 0 otherwise*), being adjacent (i.e., sharing a physical boundary) to a county with an MIP (*MIPadj = 1, 0 otherwise*) or neither. The map reports publicly available U.S. Department of Defense properties including those installations identified in Appendices 1 and 3 of Part 802 [34,35]. For each MIP, we also manually collected the date it was established. This was done to accommodate flexible indicators for MIP and MIP adjacent counties in the panel dataset, which equaled to 1 only in years where a MIP was present in the county. However, given that the majority of MIPs were established prior to 1996 [36,37], the indication is almost time invariant. If a MIP geographically spanned multiple counties, then all counties containing that point were marked as containing a military interest point, and the adjacent counties to all counties marked as containing an interest point were all marked as adjacent to a county with military interests.

Acknowledging that investors might also be seeking out agricultural land that can be considered particularly fertile, we added an additional variable, *LandValue*, to our data set capturing the average county level agricultural land value. Agricultural land values are influenced by an array of factors, including soil properties, spatial and demographic features, and market dynamics [38]. Consequently, determining the overall value requires accounting for a broad range of control variables allowing us to use it as a composite variable. Land values were gathered from Acres which has parcel details from county assessors regarding land values [20]. Importantly, land-value data are only available and published for the following publication years: 1997, 2002, 2007, 2012, 2017, and 2022. Thus, we linked the closest available land value data to the respective transaction and/or county, meaning any given transaction is assigned a land value at most 2.5 years removed from the transaction year. We acknowledge, however, that this approach can only absorb national time trends in land values via the transaction-year fixed effects and does not capture county-level changes in land markets between publication years, which is an inherent constraint of data availability. Following this we had to drop 11 transactions due to the lack of land values associated with the transactions. Ten of these were in Alaska and one was in Colorado.

Finally, we generated an indicator capturing legislation limiting land acquisitions by foreign nations (*Policy = 1, 0 otherwise*). Legislation timing is tracked using the effective date of each state's first foreign-ownership-restriction law.

### 2.3. Data analysis

Our empirical analysis is conducted on the transaction-level cross- sectional dataset. This choice reflects both the nature of our research question and the properties of our data. Our research question concerns the characteristics of individual foreign land transactions (i.e., whether a foreign transaction's proximity to an MIP predicts its origin) which is most directly estimated at the transaction level. In addition, the sparsity of PRC-linked transactions limits statistical power for county-year panel specifications with fixed effects. We therefore rely on the cross-sectional data for formal estimation and reserve the panel data for descriptive context on the geographic distribution of MIPs relative to the full universe of U.S. counties.

To investigate the relationship between the presence of MIP in a county or adjacent county and the probability that the foreign land transaction is PRC-linked or of unidentified origin, we leverage a linear probability model (LPM) with state and year fixed effects as well as a probit model as a robustness check. Because our identifying variation is cross-sectional and MIP status is effectively time-invariant within our window, our estimates should be interpreted as associational rather than causal.

The LPM can written as:


$$\begin{array}{c} Y_{ics}=\beta_{\text{1}}MIP_{C}+\beta_{\text{2}}MIPadj_{C}+\beta_{\text{3}}\text{ln}\left(LandValue_{i}\right)+\beta_{\text{4}}Policy_{ist}+\alpha_{s}+\delta_{t}+\varepsilon_{ics} \end{array}$$ (1)

where $Y_{ics}\in \{\text{0},\text{1}\}$ is a binary outcome for transaction i in county c and state s, taking value 1 if the transaction involves PRC-linked buyers excluding Smithfield, PRC-linked buyers including Smithfield, an unidentified country of origin, or a non-adversary foreign buyer. The latter serves as a placebo for our analysis. ${MIP}_{c}$ and ${MIPadj}_{c}$ are indicators equal to 1 if county c contains or is adjacent to a military installation of priority. $ln({LandValue}_{i})$ is the log of the closest-year land value for the parcel. ${Policy}_{is}$equals 1 if state s had foreign-ownership legislation in effect at the time of transaction i. $\alpha_{s}$ and $\delta_{t}$ are state and transaction-year fixed effects, and $\varepsilon_{ics}$ is the error term. Standard errors are clustered at the state level.

As a robustness check, we re-estimate the same specification as a probit model (equation 2) and report average marginal effects for direct comparability. The probit model estimates the probability of a binary outcome using the normal CDF link function [39] and can be written as:


$$\begin{array}{c} P(Y_{ics}=\text{1}|X)=\Phi (\beta_{\text{1}}MIP_{C}+\beta_{\text{2}}MIPadj_{C}+\beta_{\text{3}}\text{ln}\left(LandValue_{i}\right)+\beta_{\text{4}}Policy_{ist}+\alpha_{s}+\delta_{t}) \end{array}$$ (2)

where Φ represents the cumulative distribution function of the standard normal distribution, **X** is the vector of independent variables, and β represents the estimated coefficients [40]. Importantly, the probit specification is estimated on a slightly different effective sample relative to the LPM, as some observations are excluded due to limited outcome variation across states. As a result, these estimates should be interpreted with caution.

Given that our identifying variation is cross-sectional and MIP status is effectively time-invariant within our window, our estimates should be interpreted as associational rather than causal. A difference-in-differences design exploiting changes in MIP status is not feasible because the vast majority of MIPs in our sample are unchanged for the duration of the study. Further, county fixed effects are not feasible in this setting because MIP and MIP-adjacent status are effectively time-invariant within our sample window. As such, including them would absorb the key explanatory variables. More granular data at the county or parcel level would offer a stronger identification strategy, but the sparsity of PRC-linked transactions and the structure of AFIDA reporting prevent this with currently available data. We therefore rely on state fixed effects and acknowledge county-level heterogeneity within states as a remaining limitation and encourage future work as data improve.

We address alternative explanations for our findings in two ways: by including non-adversary foreign transactions as a placebo outcome, which would also show MIP concentration if foreign buyers generally cluster in areas near military infrastructure, and by estimating robustness specifications that drop fixed effects, drop Texas and California as the two States with the most foreign transactions, and drop the land value control (S1 Appendix Tables A5-A9 in S1 File).

The data collection and analysis complied with terms and conditions of the sources of data leveraged.

## 3. Results

### 3.1. Public attention to foreign land purchases has increased

**Fig 1:**
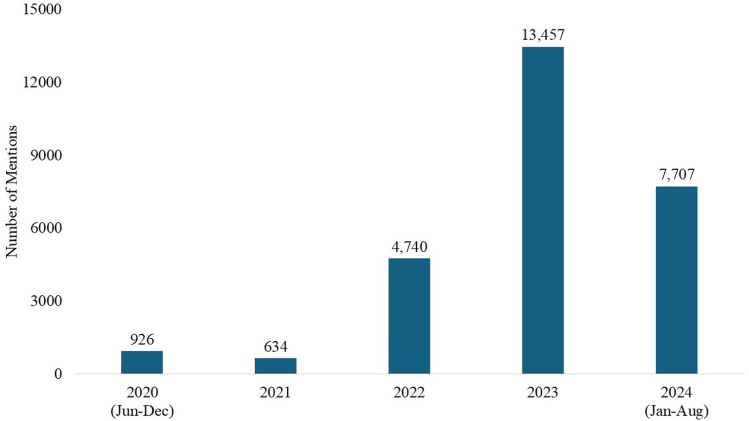
Online mentions related to foreign agricultural land holdings from June 2020-August 2024.

Fig 1 shows the yearly number of mentions of foreign agricultural land holdings in online and social media across our study period. Similar to prior work [41], mentions from Facebook and Instagram are not observed due to data restrictions [42]. While the results are presented by calendar years, we only recorded mentions for part of the year in 2020 (Week of June 14th through the end of the year) and 2024 (beginning of the year through the week of August 25th) due to the timing of our data collection. As seen, the number of recorded mentions in 2020 and 2021 are each below 1,000, but it more than quadruples in 2022, reaching 4,740 annual mentions. This increase corresponds to the introduction of different pieces of legislation for example by Representative Dan Newhouse [43]. The bill proposed by Newhouse specifically targets farmland purchases by foreign nationals associated with the PRC.

We see another substantial increase in mentions in 2023 over 2022 with 2023 amassing 13,457 total mentions. Mentions in the partial year of 2024 amount to more than 7,700, nearly doubling the 2022 total and accounting for more than half of the 2023 figure. During both 2023 and 2024 additional topical legislative proposals have been brought forth including federal ones such as Senator Tommy Tuberville’s Foreign Adversary Risk Management (FARM) Act [44] and state-based ones such as Florida’s SB 264 which restricts property ownership of persons and entities from “foreign countries of concern” [45]. Moreover, the Senate agricultural committee sought out testimony by agricultural economists on the matter [3]. In 2024, interest also grew regarding the location of farmland purchases, with several news outlets reporting on acquisitions near U.S. military bases, particularly those by PRC buyers [46,47]. As such, public attention appears to have risen contemporaneously with the state and federal legislative activity, with each major increase in mentions corresponding to concrete policy events in the same calendar year.

### 3.2. PRC-linked transactions are associated with proximity to MIPs

Across our study period, we record a total of 38,722 reported foreign transactions (see Table 1). Of these, 131 are PRC-linked, with 60 of them being attributable to the Smithfield purchase meaning we have 71 observations for PRC excluding Smithfield. 1,337 transactions are of unidentified origin. Table A4 in the Appendix S1 in S1 File reports the corresponding summary statistics for the control variables (Landvalues and Policy).

**Table 1:** Foreign transaction type summary for 1996-2024.

Transaction type	MIP present	MIP adjacent	Neither	Total
Total Foreign Transactions	4358	13550	20814	38722
PRC -linked Transactions excl. Smithfield	31	16	24	71
PRC-linked Transactions	39	47	45	131
Adversary-linked Transactions	46	49	50	145
Adversary-linked Transactions excl. PRC	7	2	5	14
Unidentified Transactions	153	539	645	1337
Non-adversarial Foreign Transactions	4160	12962	20119	37241

**Fig 2:**
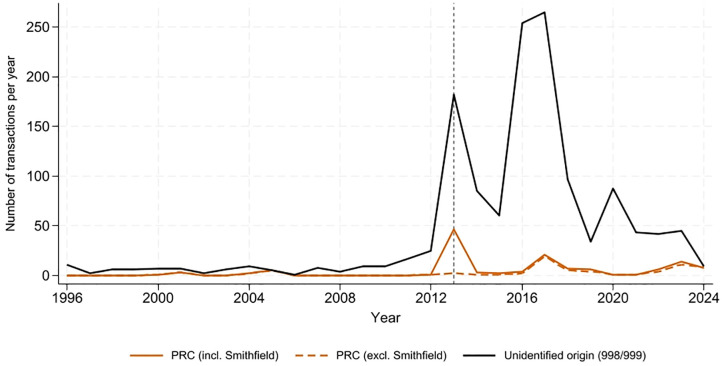
PRC and unidentified-origin transactions, 1996-2024. Note: Dashed gray line represents the WH Group-Smithfield acquisition (2013).

Fig 2 shows the annual number of PRC-linked and unidentified-origin transactions from 1996 through 2024. For PRC acquisitions excluding the large 2013 Smithfield exchange, the number of transactions remained low and fairly stable through 2016, after which we observe a modest increase. Unidentified-origin transactions show three distinct peaks in 2013, 2016–2017, and 2020.

**Fig 3:**
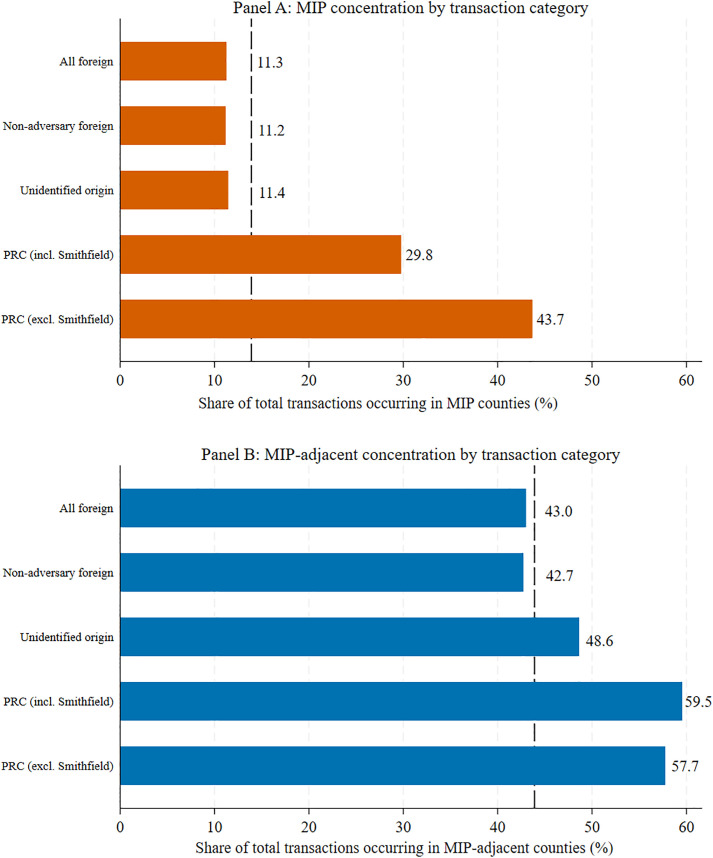
MIP and MIP adjacent concentration by transaction category, 1996-2024. Note: Dashed line represents the national county-level base rate (share of counties in the U.S. that are MIP counties [13.9%] or MIP adjacent [43.9%]).

In terms of MIP locations we find that, 13.9% of all U.S. counties contain MIPs and 43.9% of counties are adjacent to counties with MIPs. Looking at the share of transactions occurring in MIP and MIP-adjacent counties, we find that a substantially higher share of PRC-linked transactions occurs in MIP counties than is the case for all foreign or non- adversary foreign transactions (Fig 3, Panel A). PRC-linked transactions excluding Smithfield occur in MIP counties at a rate of 43.7%, compared with 11.3% for all foreign transactions. Including Smithfield reduces the PRC MIP share to 29.8%, reflecting that several Smithfield-linked parcels are in non- MIP counties, but the share remains well above the baseline for other foreign transaction categories. For MIP-adjacent counties (Fig 3, Panel B), the pattern is less stark: PRC-linked transactions excluding Smithfield occur in adjacent counties at 57.7%, compared with 43.0% for all foreign transactions and 48.6% for transactions of unidentified origin.

To examine whether these patterns are statistically notable, we estimate the LPM and probit specifications described in Section 2.3 (Table 2 and Table 3; raw probit coefficients are reported in S1 Appendix Table A8 in S1 File). Consistent with our expectations, we find no significant relationship between MIP or MIP-adjacent status and non-adversary foreign transactions in either model. No significant relationship is identified for unidentified-origin transactions either. For PRC-linked transactions excluding Smithfield, MIP presence in the same county is associated with a higher probability of PRC involvement in both the LPM (0.0047, p < 0.10) and the probit model (0.0075, p < 0.01). The effect size is small in absolute terms because PRC transactions are very few in number in the sample overall: a 0.47 percentage-point increase in the probability of PRC involvement represents a meaningful relative effect when compared against a sample-wide PRC rate of 0.18%. Higher land values are also positively and significantly associated with PRC-linked transactions. MIP-adjacency is not significantly associated with any of the outcomes in either specification. To assess the robustness of our results, we estimate additional LPM specifications: without fixed effects, with state fixed effects only, excluding Texas and California as the two states with the largest PRC-linked transaction counts, and excluding land value as a control. Results are reported in S1 Appendix Tables A5 through A9 in S1 File and are consistent with the main specifications

**Table 2:** Cross-sectional LPM – MIP proximity and foreign land transactions.

	(1)	(2)	(3)	(4)
	PRC excl. Smith	PRC incl. Smith	Unidentified	Non-adversary (placebo)
MIP	0.0047^*^	0.0041	−0.0008	−0.0044
	(0.0025)	(0.0026)	(0.0094)	(0.0100)
MIP_adj	−0.0003	0.0005	−0.0011	0.0009
	(0.0008)	(0.0012)	(0.0145)	(0.0147)
ln(Landvalue)	0.0026^**^	0.0019	0.0019	−0.0053
	(0.0012)	(0.0013)	(0.0104)	(0.0102)
Policy	0.0014	0.0014	−0.0266	0.0266
	(0.0042)	(0.0043)	(0.0206)	(0.0208)
Constant	−0.0194^**^	−0.0125	0.0218	1.0020^***^
	(0.0094)	(0.0104)	(0.0842)	(0.0831)
Observations	38721	38721	38721	38721
R-squared	0.0167	0.0385	0.0787	0.0811
Clusters (states)	47	47	47	47

Standard errors in parentheses. Standard errors clustered at state level. All regressions include State and year fixed effects. ^*^ *p* < 0.10, ^**^ *p* < 0.05, ^***^ *p* < 0.01.

**Table 3:** Panel B: Probit average marginal effects – MIP proximity and foreign land transactions.

	(1)	(2)	(3)	(4)
	PRC excl. Smith	PRC incl. Smith	Unidentified	Non-adv (placebo)
MIP	0.0075^***^	0.0064^*^	−0.0007	−0.0044
	(0.0025)	(0.0034)	(0.0084)	(0.0083)
MIP_adj	−0.0002	0.0008	−0.0016	0.0016
	(0.0017)	(0.0020)	(0.0118)	(0.0117)
ln(Landvalue)	0.0037^***^	0.0033^**^	0.0024	−0.0062
	(0.0012)	(0.0015)	(0.0087)	(0.0084)
Policy	0.0039	0.0030	−0.0280	0.0213
	(0.0045)	(0.0037)	(0.0188)	(0.0194)
Observations	15371	19765	37697	38588

Standard errors in parentheses. Standard errors clustered at state level. All regressions include State and year fixed effects. Effective N is smaller than Panel A because probit drops states with no positive outcomes. ^*^ *p* < 0.10, ^**^ *p* < 0.05, ^***^ *p* < 0.01.

Effective state legislation is not statistically significant in any of the specifications. However, because the main legislative wave began in 2022, only a small number of transactions took place under effective legislation, particularly for our adversary-linked categories. Specifically, 23 PRC-linked transactions including Smithfield, 28 excluding Smithfield, and 96 unidentified-origin transactions fall into the post-2022 window. These sample sizes are too small to identify a statistically meaningful legislative effect with our specification, and we interpret the legislation coefficient as uninformative rather than as evidence of no effect.

### 3.3. Directional evidence on pre- and post-2022 patterns

**Fig 4:**
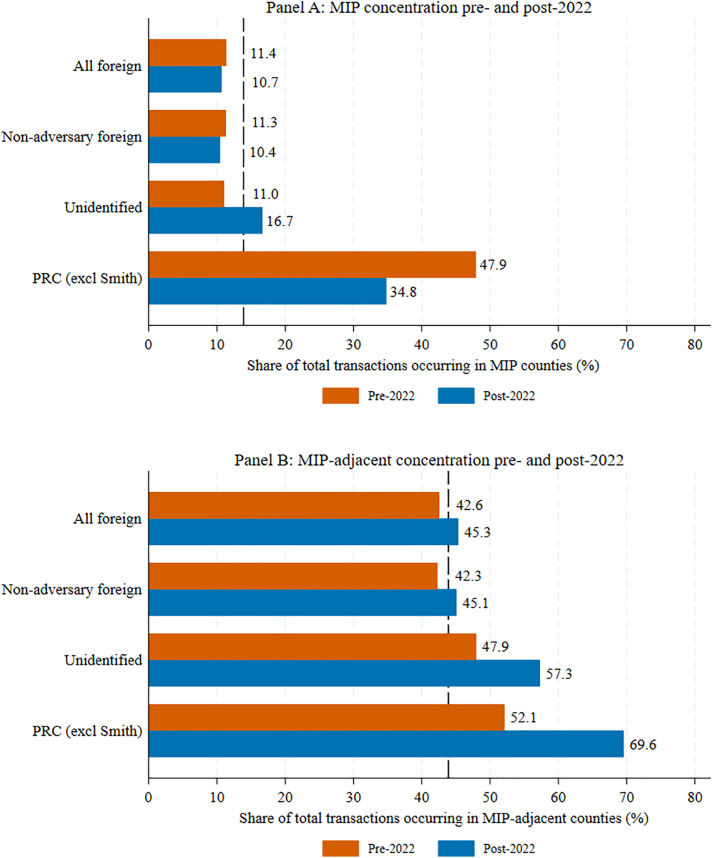
MIP and MIP adjacent Concentration by category, pre- and post 2022. Note: Dashed line represents the national county-level base rate (share of counties in the U.S. that are MIP counties [13.9%] or MIP adjacent [43.9%]).

Given the above-mentioned sample-size constraints, we look descriptively at how the spatial distribution of foreign transactions differs between the pre- 2022 period (1996−2021) and the post-2022 period (2022−2024). Fig 4 reports the share of transactions in MIP counties (Panel A) and MIP- adjacent counties (Panel B) for each outcome category, split by period. Since the window is only three years long, post-2022 transaction counts are small and AFIDA reporting lags may affect comparability across periods. As such, these shares are descriptive and should not be interpreted as causal effects of the legislative wave.

We find that for PRC-linked transactions excluding Smithfield, the MIP-county share fell from 47.9% before 2022 to 34.8% after 2022, a decline of 13.1%, while the MIP- adjacent share rose from 52.1% to 69.6%, an increase of 17.5%. While they are substantial, the small number of post-2022 PRC transactions (n = 28) means this pattern could equally reflect sampling variation. For unidentified-origin transactions, both the MIP-county share (+5.7%) and the MIP-adjacent share (+9.4%) rose post-2022. This is consistent with a shift toward opaque ownership structures coinciding with the legislative wave, though the same sample-size caveats apply. Meanwhile, the placebo category of non-adversary foreign transactions shows no meaningful change across either MIP or MIP-adjacent shares between periods (−0.9% for MIP; + 1.8% for MIP-adjacent). This supports the interpretation that the shifts observed for PRC and unidentified-origin transactions are not driven by general trends in foreign investment patterns.

## 4. Discussion and conclusion

Our results document growing public interest in foreign agricultural land holdings, occurring alongside increased attention from policymakers. The initial growth in interest is often attributed to the highly publicized purchase of 130,000 acres near a U.S. Air Force Base by a PRC-owned company as well as the purchase of 300 acres by the Fufeng Group, a Chinese company, near another Air Force Base in North Dakota [48]. Indeed, since 2021 more than 22 states have brought forth legislation to curtail foreign land holdings [7]. Likewise, several different pieces of legislation were introduced during the 118th Congress that focus on restricting land purchases by foreign entities, in particular by adversarial nations, including seven proposals that aim to add agriculture under the jurisdiction of CFIUS [49]. Thus, it appears that the matter is growing in regulatory and public policy discourse, albeit sustained interest in the matter cannot be ensured.

Previous studies and expert testimonies on the matter have mostly dismissed concerns of purchases and leases of farm land by foreign adversarial nations [3,50] due to the small share of land actually held by these countries. However, our results suggest a statistical relationship between land-holdings by adversaries and the proximity of land to U.S. MIPs. As such, our results could lend support to President Joe Biden's 2024 proposal that CFIUS should review land sales to foreign investors when the property is located within 100 miles of eight specific U.S. military bases [51]. As well as proposals brought forth by Republicans in the subsequent administration including the Not One More Inch or Acre Act led by Tom Cotton [52]. Our findings also complement Lin and Ortega's [8] analysis of legislative drivers: where their work shows that legislators respond to military installations and Chinese investment when crafting restrictions, our results document that the underlying spatial concentration they reference is empirically present in the transaction data.

We emphasize, however, that our estimates are associational rather than causal, and the effect sizes are small in absolute terms. A 0.47 percentage point to 0.75 percentage point increase in the probability that a transaction is PRC-linked, against a sample-wide PRC rate below 0.2%, represents a meaningful relative concentration but a small absolute number of transactions in MIP counties. Our results therefore inform, but do not settle, the debate about whether the spatial pattern of foreign land acquisitions warrants the regulatory response currently under discussion.

Our results should be considered within the framework of the existing data limitations. For example, to identify MIPs we solely relied on easily accessible public data on geographical locations of domestic U.S. military bases. As such, we did not consider facilities that are not listed in the CFIUS map but still hold national security relevance. Further, county-level analysis may also mask finer spatial variation within counties: parcels immediately adjacent to military base boundaries are treated identically to parcels on the far side of MIP or MIP-adjacent counties. This aggregation likely attenuates our estimated MIP coefficients toward zero, implying that the true spatial concentration of PRC-linked acquisitions near military installations may be stronger than our estimates indicate.

A related limitation concerns the granularity of our legislation indicator. The Policy variable captures whether state-level foreign- ownership legislation was in effect at the time of a transaction, but does not distinguish between country-neutral provisions (which apply to all foreign entities) and country-specific provisions that target acquisitions by particular adversarial nations such as the PRC (for example, South Dakota’s 2024 HB1231 [53]). To the extent that country-specific laws generate different compliance responses than country-neutral laws, pooling them into a single indicator may mask heterogeneous effects. Given the small number of PRC-linked transactions occurring under any form of effective legislation (n = 28 post-2022 excluding Smithfield), our data do not support separate identification of country-specific legislative effects. This is an important direction for future research as additional post-2022 transactions are reported and as more states enact PRC-specific provisions.

Even more importantly, the AFIDA data used is subject to a host of short-comings as also recently pointed out by a report of the Government Accountability Office [30] and acknowledged in the AFIDA report for 2024 [2]. To begin with, the most recent AFIDA data set available is from 2024, meaning transactions since then have not been reported, which is particularly pertinent given the above-mentioned increase in foreign land-holdings by about 2.2 million acres per year [4]. Due to established procedures, notifications about foreign land-holdings have to be made on paper forms leading to delays in reporting [30]. Although the mandate to develop an online submission form has been given, corresponding funds have not yet been allocated, and no timeline has been developed for this process. Further compounding this issue is the current lack of capacity to monitor and reprimand non-compliance. While initial efforts have been made in the form of data mining, progress is stagnant due to weak internal processes [30].

Relatedly, the available data only recently began recording secondary investors for adversarial nations [2]. As noted in our results, a nontrivial share of transactions cannot be linked to a clear country of origin when no foreign investor is listed or when multiple countries appear on the reporting form without an easily identifiable primary shareholder [2]. Because of this, the 2024 AFIDA report states that “*the acreage associated with the [PRC] investors—or any other country discussed in this report—should be interpreted as a minimum*” [2]. Given our descriptive finding that the share of unidentified-origin transactions rose in MIP and MIP-adjacent counties post-2022, opaque ownership structures may be an increasing challenge for attribution over time. Future research might try to disentangle these structures to facilitate a clearer assessment of spatial patterns.

Of further relevance is the current regulation about how land leased by foreign entities has to be reported to and is captured by AFIDA. Taylor, Zhang and Attah [13] highlighted that a large share of the land held by foreign entities is being leased rather than owned, a trend that has accelerated in recent years. However, leases of land are recorded if leaseholds are 10 years or more [2], meaning that shorter leases might exist, but are not reported or recorded. Taken together, our measured effects thus likely represent lower bounds.

Our descriptive pre- and post-2022 evidence should be interpreted with caution. Only three years of post-2022 data are available, post-2022 transaction counts for our adversary-linked categories are small (28 PRC-linked transactions excluding Smithfield, and 96 unidentified-origin transactions), and AFIDA reporting lags are most pronounced for the most recent years. Nevertheless, we observe a decline in the share of PRC-linked transactions in MIP counties alongside an increase in MIP-adjacent counties, and a rise in the MIP and MIP-adjacent shares of unidentified-origin transactions. These patterns are directionally consistent with a scenario in which heightened regulatory attention reshapes where and under what ownership structures transactions occur, but the small sample sizes preclude statistical inference. We report these patterns to document the data as they stand and to motivate continued monitoring as additional post-2022 data become available, not as evidence of a causal policy effect.

Beyond AFIDA limitations, additional context is needed for interpreting our findings. There are documented cases of malicious actions being taken against the US, including hacking of critical infrastructure by the PRC [54,55] or drone incursions of military bases [56,57] which further highlights the need to understand the exact uses of lands held by foreign adversarial countries within the US. At the same time, we do not observe the transactions of agricultural land holdings by adversaries near other strategic assets such as power or water management facilities or large data centers, which represents an additional gap in the current evidence base.

We also want to note that our analysis deliberately focuses on transaction counts rather than acreage acquired or the underlying transaction value, given that our focus lies on whether transactions linked to the PRC, unidentified ownership, or non-adversary foreign buyers are spatially concentrated near military interest points. In this context, the presence and location of a transaction are important because potential national-security concerns may arise even from small parcels, depending on their proximity to sensitive infrastructure and their ultimate use. Indeed, that the President blocked an attempt to purchase 17 acres of land near a military base in Wyoming by a PRC-majority-owned cryptocurrency company in 2024 highlights that the size of the acquisition is less relevant [58,59]. Acreage- or transaction-value-weighted outcomes would instead place greater emphasis on the scale or economic magnitude of acquisitions, which is an important but distinct question. Such measures may be especially useful for future research that could assess whether adversary-linked or opaque foreign buyers acquire larger quantities of land, higher-value land, or parcels that may affect local agricultural land markets.

While foreign investment in U.S. agricultural land has been previously discussed in the context of agricultural markets, food security, land prices, and other food or farming oriented reasons, the conversation about holdings of U.S. farmland for non-agricultural reasons is sparse. This analysis provides evidence that foreign farmland holdings are statistically related to the location of U.S. MIPs when analyzed at the county level. Our findings are relevant to ongoing policy discussions regarding the scope of land‑transaction review mechanisms, including proposals to expand geographic screening near military installations. It follows that regulation and funding for tracking and further study may need to encompass multiple government agencies as this analysis has provided evidence that farmland ownership may be motivated by multiple drivers, that may extend beyond proximity to MIPs, originating within and outside of agricultural interests.

## Supporting information

S1 File Appendix includes Table A1-A9. (DOCX)

S2 File Social Media Listening Data. (XLSX)

S3 File Cross-Sectional Data. (DTA)

S4 File Panel Data. (DTA)
