# Supplementary material for: Spatial patterns of foreign agricultural land holdings near U.S. military interest points
Source: PLoS One. 2026 Aug 12;21(8):e0355827. doi: 10.1371/journal.pone.0355827 (PMC13465803; doi:10.1371/journal.pone.0355827)
Supplement: Supplementary file 1 [file pone.0355827.s001.docx]

**Table A1. Inclusionary Search Terms for Social Listening.**

| **General Search** | | | | | | | | |
| --- | --- | --- | --- | --- | --- | --- | --- | --- |
| foreign-owned land | | Chinese-owned land | Russian-owned land | Iranian-owned land | North Korean-owned land | Cuban-owned land | Venezuelan-owned land | |
| foreign-held land | | Chinese-held land | Russian-held land | Iranian-held land | North Korean-held land | Cuban-held land | Venezuelan-held land | |
| foreign land holdings | | Chinese land holdings | Russian land holdings | Iranian land holdings | North Korean land holdings | Cuban land holdings | Venezuelan land holdings | |
| foreign land investments | | Chinese land investments | Russian land investments | Iranian land investments | North Korean land investments | Cuban land investments | Venezuelan land investments | |
| foreign land interests | | Chinese land interests | Russian land interests | Iranian land interests | North Korean land interests | Cuban land interests | Venezuelan land interests | |
| foreign-owned farms | | Chinese-owned farms | Russian-owned farms | Iranian-owned farms | North Korean-owned farms | Cuban-owned farms | Venezuelan-owned farms | |
| foreign demand for land | | Chinese demand for land | Russian demand for land | Iranian demand for land | North Korean demand for land | Cuban demand for land | Venezuelan demand for land | |
| foreign Ownership of U.S. Agriculture | | Chinese Ownership of U.S. Agriculture | Russian Ownership of U.S. Agriculture | Iranian Ownership of U.S. Agriculture | North Korean Ownership of U.S. Agriculture | Cuban Ownership of U.S. Agriculture | Venezuelan Ownership of U.S. Agriculture | |
| AFIDA |  | Chinese-held ag land | Russian-held ag land | Iranian-held ag land | North Korean-held ag land | Cuban-held ag land | Venezuelan-held ag land | |
| foreign-held ag land | | Chinese agriculture investment | Russian agriculture investment | Iranian agriculture investment | North Korean agriculture investment | Cuban agriculture investment | Venezuelan agriculture investment | |
| Foreign agriculture investment | | Chinese Ownership of farmland | Russian Ownership of farmland | Iranian Ownership of farmland | North Korean Ownership of farmland | Cuban Ownership of farmland | Venezuelan Ownership of farmland | |
| foreign Ownership of farmland | | Chinese land acquisition | Russian land acquisition | Iranian land acquisition | North Korean land acquisition | Cuban land acquisition | Venezuelan land acquisition | |
| Agricultural Foreign Investment Disclosure Act | | Chinese agricultural land acquisition | Russian agricultural land acquisition | Iranian agricultural land acquisition | North Korean agricultural land acquisition | Cuban agricultural land acquisition | Venezuelan agricultural land acquisition | |
| foreign land acquisition | | Chinese agland acquisition | Russian agland acquisition | Iranian agland acquisition | North Korean agland acquisition | Cuban agland acquisition | Venezuelan agland acquisition | |
| foreign agricultural land acquisition | | Chinese land ownership restrictions | Russian land ownership restrictions | Iranian land ownership restrictions | North Korean land ownership restrictions | Cuban land ownership restrictions | Venezuelan land ownership restrictions | |
| foreign agland acquisition | | Chinese Ownership of U.S. Agricultural land | Russian Ownership of U.S. Agricultural land | Iranian Ownership of U.S. Agricultural land | North Korean Ownership of U.S. Agricultural land | Cuban Ownership of U.S. Agricultural land | Venezuelan Ownership of U.S. Agricultural land | |
| foreign land ownership restrictions | | Chinese agricultural leases | Russian agricultural leases | Iranian agricultural leases | North Korean agricultural leases | Cuban agricultural leases | Venezuelan agricultural leases | |
| foreign Ownership of U.S. Agricultural land | foreign landholders | Agricultural Foreign Investment Disclosure | Prohibition of Foreign Agricultural Land Ownership | | | | |  |
| foreign agricultural leases | Foreign land holdings | foreign private investment in agricultural land | Prohibition of Foreign Agricultural Land Investment | | | | |  |
| Foreign ownership of agricultural land | Agricultural Foreign Investment | bans on ag land purchases | |  |  |  |  |  |

**Table A2. Exclusionary Search Terms for Social Listening.**

**Excluded Terms**

Afida Turner

Ronnie turner

Ronnie Turner

Ronnie's Widow

Ronnie's widow

Wife Afida

wife Afida

wife afida

ronnie turner

afida turner

afida Turner

French media personality Afida

Australian homes

**Table A3. Domains (from Quid search).**

| **Partial 2020** | | **2020-2021** | | **2021-2022** | | **2022-2023** | | **2023-2024** | |
| --- | --- | --- | --- | --- | --- | --- | --- | --- | --- |
| twitter.com | 100 | twitter.com | 820 | twitter.com | 1190 | twitter.com | 8510 | twitter.com | 5440 |
| boards.4chan.org | 5 | boards.4chan.org | 34 | boards.4chan.org | 46 | nationalaglawcenter.org | 122 | law360.com | 797 |
| agriculture.com | 5 | namepros.com | 15 | investigatemidwest.org | 32 | newsbreak.com | 90 | ruralradio.com | 126 |
| lipstickalley.com | 4 | euronews.biz | 10 | ar15.com | 30 | farmprogress.com | 75 | foreignaffairs.co.nz | 102 |
| gamefaqs.gamespot.com | 4 | boards.4channel.org | 9 | wcax.com | 26 | spamchronicles.com | 51 | newsbreak.com | 85 |
| edition.cnn.com | 2 | farmworldonline.com | 8 | ksfy.com | 25 | agri-pulse.com | 42 | nationalaglawcenter.org | 71 |
| furtherafrica.com | 2 | adebayothevoice.com | 8 | hawaiinewsnow.com | 25 | kttn.com | 33 | newsfromthestates.com | 68 |
| ten15am.org | 2 | farmlandgrab.org | 7 | wbrc.com | 25 | radio.wcmu.org | 32 | agri-pulse.com | 65 |
| the-latest.news | 2 | joplinglobe.com | 6 | agri-pulse.com | 24 | jdsupra.com | 31 | investigatemidwest.org | 62 |
| sheepcentral.com | 2 | raynetoday.com | 5 | kgns.tv | 23 | thehamdenjournal.com | 31 | jdsupra.com | 50 |

**Table A4. Summary statistics for control variables**

| **Control Variable** | **MIP present** | **MIP adjacent** | **Neither** | **Total** |
| --- | --- | --- | --- | --- |
| ln(Landvalue) | 7.83 | 7.96 | 7.89 | 7.91 |
| Share of counties affected by restrictive policy (%) | 4.04% | 3.73% | 10.91% | 7.60% |

**Table A5. LPM without fixed effects.**

|  | (1) | (2) | (3) | (4) |
| --- | --- | --- | --- | --- |
|  | PRC excl. Smith | PRC incl. Smith | Unidentified | Non-adv (placebo) |
| mip_any | 0.0060^**^ | 0.0060^**^ | -0.0023 | -0.0049 |
|  | (0.0023) | (0.0024) | (0.0085) | (0.0088) |
|  |  |  |  |  |
| mip_adj_any | 0.0003 | 0.0014 | 0.0060 | -0.0072 |
|  | (0.0006) | (0.0016) | (0.0120) | (0.0122) |
|  |  |  |  |  |
| log_landvalue | 0.0018^*^ | 0.0018 | 0.0019 | -0.0040 |
|  | (0.0009) | (0.0011) | (0.0052) | (0.0057) |
|  |  |  |  |  |
| leg_effective | 0.0002 | -0.0009 | -0.0300^***^ | 0.0314^***^ |
|  | (0.0016) | (0.0021) | (0.0073) | (0.0078) |
|  |  |  |  |  |
| _cons | -0.0130^*^ | -0.0122 | 0.0191 | 0.9944^***^ |
|  | (0.0068) | (0.0082) | (0.0446) | (0.0481) |
| Observations | 38722 | 38722 | 38722 | 38722 |
| R-squared | 0.0030 | 0.0019 | 0.0022 | 0.0025 |
| Clusters (states) | 48 | 48 | 48 | 48 |

Standard errors in parentheses

Same sample and controls as Table 2 Panel A but without state or year FE.

^*^ *p* < 0.10, ^**^ *p* < 0.05, ^***^ *p* < 0.01

**Table A6. LPM with state FE only.**

|  | (1) | (2) | (3) | (4) |
| --- | --- | --- | --- | --- |
|  | PRC excl. Smith | PRC incl. Smith | Unidentified | Non-adv (placebo) |
| mip_any | 0.0049^*^ | 0.0039 | 0.0001 | -0.0050 |
|  | (0.0025) | (0.0026) | (0.0100) | (0.0107) |
|  |  |  |  |  |
| mip_adj_any | -0.0003 | 0.0002 | 0.0010 | -0.0008 |
|  | (0.0007) | (0.0011) | (0.0138) | (0.0137) |
|  |  |  |  |  |
| log_landvalue | 0.0027^**^ | 0.0018 | 0.0062 | -0.0089 |
|  | (0.0012) | (0.0012) | (0.0073) | (0.0073) |
|  |  |  |  |  |
| leg_effective | 0.0039 | 0.0049 | -0.0357^*^ | 0.0324 |
|  | (0.0043) | (0.0043) | (0.0212) | (0.0212) |
|  |  |  |  |  |
| _cons | -0.0202^**^ | -0.0114 | -0.0123 | 1.0307^***^ |
|  | (0.0091) | (0.0099) | (0.0578) | (0.0580) |
| Observations | 38721 | 38721 | 38721 | 38721 |
| R-squared | 0.0130 | 0.0283 | 0.0369 | 0.0354 |
| Clusters (states) | 47 | 47 | 47 | 47 |

Standard errors in parentheses

Same sample and controls as Table 2 Panel A but without year FE.

^*^ *p* < 0.10, ^**^ *p* < 0.05, ^***^ *p* < 0.01

**Table A7. LPM excluding Texas and California.**

|  | (1) | (2) | (3) | (4) |
| --- | --- | --- | --- | --- |
|  | PRC excl. Smith | PRC incl. Smith | Unidentified | Non-adv (placebo) |
| mip_any | 0.0028^*^ | 0.0022 | 0.0048 | -0.0080 |
|  | (0.0016) | (0.0017) | (0.0091) | (0.0097) |
|  |  |  |  |  |
| mip_adj_any | -0.0003 | 0.0005 | -0.0028 | 0.0023 |
|  | (0.0008) | (0.0013) | (0.0166) | (0.0168) |
|  |  |  |  |  |
| log_landvalue | 0.0023 | 0.0012 | 0.0122 | -0.0144 |
|  | (0.0015) | (0.0017) | (0.0099) | (0.0104) |
|  |  |  |  |  |
| leg_effective | 0.0021 | 0.0019 | -0.0328 | 0.0320 |
|  | (0.0040) | (0.0042) | (0.0206) | (0.0208) |
|  |  |  |  |  |
| _cons | -0.0178 | -0.0069 | -0.0601 | 1.0753^***^ |
|  | (0.0116) | (0.0135) | (0.0829) | (0.0875) |
| Observations | 33952 | 33952 | 33952 | 33952 |
| R-squared | 0.0181 | 0.0478 | 0.0896 | 0.0929 |
| Clusters (states) | 45 | 45 | 45 | 45 |

Standard errors in parentheses

These two states account for 44% of PRC transactions.

^*^ *p* < 0.10, ^**^ *p* < 0.05, ^***^ *p* < 0.01

**Table A8. LPM without land value control.**

|  | (1) | (2) | (3) | (4) |
| --- | --- | --- | --- | --- |
|  | PRC excl. Smith | PRC incl. Smith | Unidentified | Non-adv (placebo) |
| mip_any | 0.0048^*^ | 0.0042 | -0.0007 | -0.0045 |
|  | (0.0025) | (0.0026) | (0.0095) | (0.0101) |
|  |  |  |  |  |
| mip_adj_any | -0.0001 | 0.0006 | -0.0010 | 0.0006 |
|  | (0.0008) | (0.0012) | (0.0148) | (0.0150) |
|  |  |  |  |  |
| leg_effective | 0.0018 | 0.0017 | -0.0263 | 0.0257 |
|  | (0.0042) | (0.0042) | (0.0204) | (0.0205) |
|  |  |  |  |  |
| _cons | 0.0012^*^ | 0.0025^***^ | 0.0370^***^ | 0.9600^***^ |
|  | (0.0006) | (0.0007) | (0.0066) | (0.0066) |
| Observations | 38721 | 38721 | 38721 | 38721 |
| R-squared | 0.0161 | 0.0383 | 0.0787 | 0.0809 |
| Clusters (states) | 47 | 47 | 47 | 47 |

Standard errors in parentheses

Same as Table 2 Panel A without log(land value).

^*^ *p* < 0.10, ^**^ *p* < 0.05, ^***^ *p* < 0.01

**Table A9: Probit coefficient estimates.**

|  | (1) | (2) | (3) | (4) |
| --- | --- | --- | --- | --- |
|  | PRC excl. Smith | PRC incl. Smith | Unidentified | Non-adv (placebo) |
| main |  |  |  |  |
| mip_any | 0.7016^***^ | 0.4735^*^ | -0.0116 | -0.0647 |
|  | (0.2381) | (0.2509) | (0.1309) | (0.1214) |
|  |  |  |  |  |
| mip_adj_any | -0.0222 | 0.0580 | -0.0243 | 0.0234 |
|  | (0.1544) | (0.1467) | (0.1846) | (0.1718) |
|  |  |  |  |  |
| log_landvalue | 0.3466^***^ | 0.2452^**^ | 0.0372 | -0.0903 |
|  | (0.1149) | (0.1118) | (0.1370) | (0.1239) |
|  |  |  |  |  |
| leg_effective | 0.3654 | 0.2227 | -0.4381 | 0.3118 |
|  | (0.4215) | (0.2771) | (0.2951) | (0.2851) |
| Observations | 15371 | 19765 | 37697 | 38588 |

Standard errors in parentheses

Raw probit coefficients (not marginal effects; see Table 2 Panel B for AMEs).

Same specifications as Panel B: includes state and year FE, clustered SEs.

State and year fixed-effect coefficients suppressed for readability.

^*^ *p* < 0.10, ^**^ *p* < 0.05, ^***^ *p* < 0.01
